# Identification of key circadian rhythm genes in skin aging based on bioinformatics and machine learning

**DOI:** 10.18632/aging.205155

**Published:** 2023-10-30

**Authors:** Xiao Xiao, Hao Feng, Yangying Liao, Hua Tang, Lan Li, Ke Li, Feng Hu

**Affiliations:** 1 Department of Dermatology, Hunan Provincial People’s Hospital (The First-Affiliated Hospital of Hunan Normal University), Changsha, Hunan 410002, China

**Keywords:** skin aging, circadian rhythm, immune infiltration, bioinformatics, machine learning

## Abstract

Skin aging is often accompanied by disruption of circadian rhythm and abnormal expression of circadian rhythm-related genes. In this study, we downloaded skin aging expression datasets from the GEO database and utilized bioinformatics and machine learning methods to explore circadian rhythm genes and pathways involved in skin aging, revealing the pathological and molecular mechanisms of skin aging. Results showed that 39 circadian rhythm-related genes (CRGs) were identified in skin aging, and these CRGs were enriched in signaling pathways such as glucagon signaling pathway, insulin resistance, thyroid hormone signaling pathway, and adipocytokine signaling pathway. Three key skin aging-related CRGs, SIRT1, ARNTL, and ATF4, were identified based on machine learning. Additionally, we found that skin aging was associated with infiltration of immune cells including NK cells activated, Macrophages M1, Mast cells resting, T cells CD4 memory activated, and Macrophages M2, and the expression of the three key skin aging-related CRGs was correlated with these immune cells. Finally, SIRT1, ARNTL, and ATF4 were all down-regulated in skin aging and had a good ability to distinguish young skin tissue from aging skin tissue. In conclusion, three key CRGs, including SIRT1, ARNTL, and ATF4, which are closely related to skin aging, were obtained based on bioinformatics and machine learning technology screening. These three key CRGs were potential risk genes for skin aging and also associated with changes in the immune microenvironment in skin aging. The language used in this paragraph follows the guidelines for scientific writing specified by SCI, making it clear and concise.

## INTRODUCTION

Aging is a normal and complex biological process characterized primarily by progressive decline in tissue structure and physiological function of organisms [1]. The skin is the largest organ of the body and the outermost protective layer, which will show aging symptoms such as wrinkles, hyperpigmentation, roughness, and sagging as the organism ages. Physiological features of skin aging include epidermis thinning, elastic tissue degeneration, melanocyte loss, and barrier function impairment [2]. In recent years, with the increase of human life expectancy, the arrival of an aging society and the increase of public awareness of beauty, the problem of skin aging has attracted more and more attention, and exploring the causes of skin aging and how to delay it has become a hot spot in the field of aesthetic medicine. Moreover, skin aging is closely related to many systems of the body, and dysfunction of these systems can lead to various diseases such as skin cancer, vascular diseases, and metabolic diseases [3]. Therefore, resistance to skin aging can delay or prevent the occurrence and development of certain degenerative diseases.

Skin aging is caused by many factors, mainly divided into extrinsic and intrinsic factors. Extrinsic skin aging is mainly caused by external factors such as ionizing and non-ionizing radiation, air pollution, natural harmful gases (such as ozone and high concentrations of oxygen), smoking, microbial invasion, and viruses [4]. Ultraviolet radiation is one of the major environmental factors causing skin damage and aging [5]. In addition, age also has a significant impact on skin physiology because it results in a decline in body function such as a decrease in collagen synthesis, destruction of elastic fibers, and loss of subcutaneous fat [6]. Various factors that cause skin aging exert multiple effects through different reaction pathways, including oxidative stress, inflammation, and DNA damage. These factors may not be limited to the concept of skin aging and may also lead to the occurrence and development of severe diseases such as skin cancer, diabetes, cardiovascular disease, and autoimmune diseases [6].

Circadian rhythm is a natural biological clock within the human body that mainly operates with a 24-hour periodicity. Circadian rhythm consists of a central oscillator and peripheral oscillators, regulated by a transcription-translation feedback loop formed by rhythm genes [7]. The central oscillator is located in the pineal gland in the cerebral cortex and hypothalamus of the brain, and it mainly controls the periodicity of the entire circadian rhythm by perceiving changes in light, thereby regulating the physiological and behavioral changes of the human body such as sleep, diet, and mental state. The central oscillator has high synchronicity and can synchronize environmental light changes through neural transmission to peripheral oscillators (multiple peripheral tissues and organs, including skin, liver, kidneys, lungs, heart, etc.) [8]. As a peripheral oscillator, the skin is controlled by the central oscillator and has autonomous rhythms [9]. Studies have shown that skin aging is related to abnormal expression of rhythm genes [10].

In recent years, bioinformatics and machine learning techniques have been widely used in the analysis of gene and protein expression profiles, which can help to quickly and accurately discover the molecular mechanisms under specific pathological changes. Therefore, this study used bioinformatics and machine learning techniques to explore circadian rhythm regulatory genes and molecular mechanisms that are closely associated with skin aging. Our study aimed to understand the role and mechanism of the internal clock system in regulating skin physiological status in depth and to provide new ideas and means for exploring the mechanism of skin aging.

## MATERIALS AND METHODS

### Microarray data

Transcriptomic datasets related to skin aging were downloaded from the Gene Expression Omnibus (GEO) database (https://www.ncbi.nlm.nih.gov/geoprofiles/). Three datasets were selected for analysis: GSE85358, GSE120783, and GSE39170. The GSE85358 dataset, based on the GPL17077 platform (Agilent-039494 SurePrint G3 Human GE v2 8x60K Microarray), included 24 skin tissue samples from young volunteers (Young group) and 24 skin tissue samples from elderly individuals (Old group), and was used as the training set in this study. The GSE120783 dataset, based on the GPL18573 platform (Illumina NextSeq 500), consisted of 17 normal skin tissue samples and 17 skin tissue samples treated with glucocorticoids, a commonly used anti-aging and anti-inflammatory drug in dermatology. This dataset was used as a validation set. In addition, the GSE39170 dataset, based on the GPL9115 platform (Illumina Genome Analyzer II), contained 5 skin tissue samples from young individuals and 5 skin tissue samples from old individuals, and was also used for validation. 260 genes related to the circadian rhythm (CRGs) were downloaded from the MSigDB database (Supplementary Table 1).

### Differential expression analysis and WGCNA analysis

Differential expression analysis was performed using the R package “limma” on the GSE85358 dataset to identify genes that were differentially expressed between the old and young groups. Weighted gene co-expression network analysis (WGCNA) was performed using the R package “WGCNA” to identify genes closely related to skin aging. Specifically, we constructed an adjacency matrix based on the expression matrix of differentially expressed genes (DEGs) to describe the strength of the correlation between nodes. An appropriate soft-thresholding value (β) was calculated, and the adjacency matrix was transformed into a topological overlap matrix (TOM). Hierarchical clustering and dynamic tree cut algorithms were used to identify modules. Module eigengenes (MEs), which represent the first principal component of a module, were used to calculate the correlation between the modules and clinical data to identify modules significantly associated with aging (*p*-value < 0.05). Gene significance (GS) and module membership (MM) were calculated for each module. Hub genes that were significantly associated with clinical traits (Skin aging) were selected from the co-expressed gene modules using the threshold “GS > 0.5 and MM > 0.5 and *p*-value < 0.05”.

### Selection of circadian rhythm genes associated with skin aging

Hub genes identified from the WGCNA network were compared with genes related to the circadian rhythm downloaded from the MSigDB database. The overlapping genes were selected as the circadian rhythm genes associated with skin aging (Skin aging related-CRGs).

### Functional enrichment analysis

Gene set enrichment analysis (GSEA) was performed to analyze the enrichment of circadian rhythm-related signaling pathways and biological processes between the Old and Young groups in the GSE85358 dataset using GSEA version 4.0.1 software with KEGG gene sets and GO biological processes gene sets as reference genomes. Enrichment terms with a *p*-value < 0.05 were considered significant.

The hub genes and skin aging related-CRGs were subjected to GO functional annotation and KEGG functional enrichment analysis using the DAVID database. GO functional annotation involved biological processes (BPs), cellular components (CCs), and molecular functions (MFs). Enrichment terms with a *p*-value < 0.05 were considered significant.

### Protein-protein interaction (PPI) analysis

The skin aging related-CRGs were uploaded to the STRING database (https://string-db.org/) for protein-protein interaction (PPI) analysis. The PPI network was visualized using Cytoscape software.

### Screening for key CRGs by machine learning

Two machine learning algorithms, namely LASSO regression analysis and SVM-REF analysis, were employed in this study. The gene expression matrix of skin aging related CRGs was used for the machine learning analysis. Firstly, the key genes were selected based on the LASSO regression algorithm using the R package “rms”. Then, the SVM-REF analysis was performed using the R package “e1071”. The intersection of the genes obtained from both analyses were considered as key circadian rhythm genes that are associated with skin aging.

### Immune infiltration analysis

The impact of the immune microenvironment on skin aging was investigated by analyzing the GSE85358 dataset using the CIBERSORTx database (https://cibersortx.stanford.edu/). The gene expression data from each sample were uploaded to the CIBERSORTx database to quantify the infiltration level of 22 immune cell types. We compared the infiltration levels of 22 types of immune cells in skin tissues between the young and old groups using Wilcoxon rank-sum test. Correlations were then examined between immune cells and key CRGs.

### Expression of key CRGs

The Wilcoxon rank-sum test was utilized to analyze the expression levels of the identified key CRGs. First, we measured the expression levels of key CRGs in the young and old groups of dataset GSE85358. Subsequently, these profiles were validated in the independent datasets GSE120783 and GSE39170.

### Receiver operating characteristic (ROC) analysis

To validate the accuracy of the screened key CRGs and assess the capability of key CRGs to distinguish between aging and young skin, we conducted receiver operating characteristic (ROC) analysis on the training set (GSE85358) and validation sets (GSE120783 and GSE39170), and determined the area under the curve (AUC) values using the “pROC” package.

## RESULTS

### Screening for genes associated with skin aging

**Figure 1:**
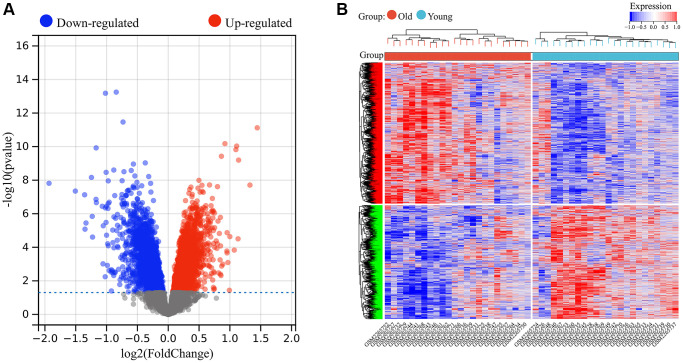
**Identification of differentially expressed genes in skin aging.** (**A**) Volcanic map of differential expression analysis. (**B**) The expression heat map of DEGs.

**Figure 2:**
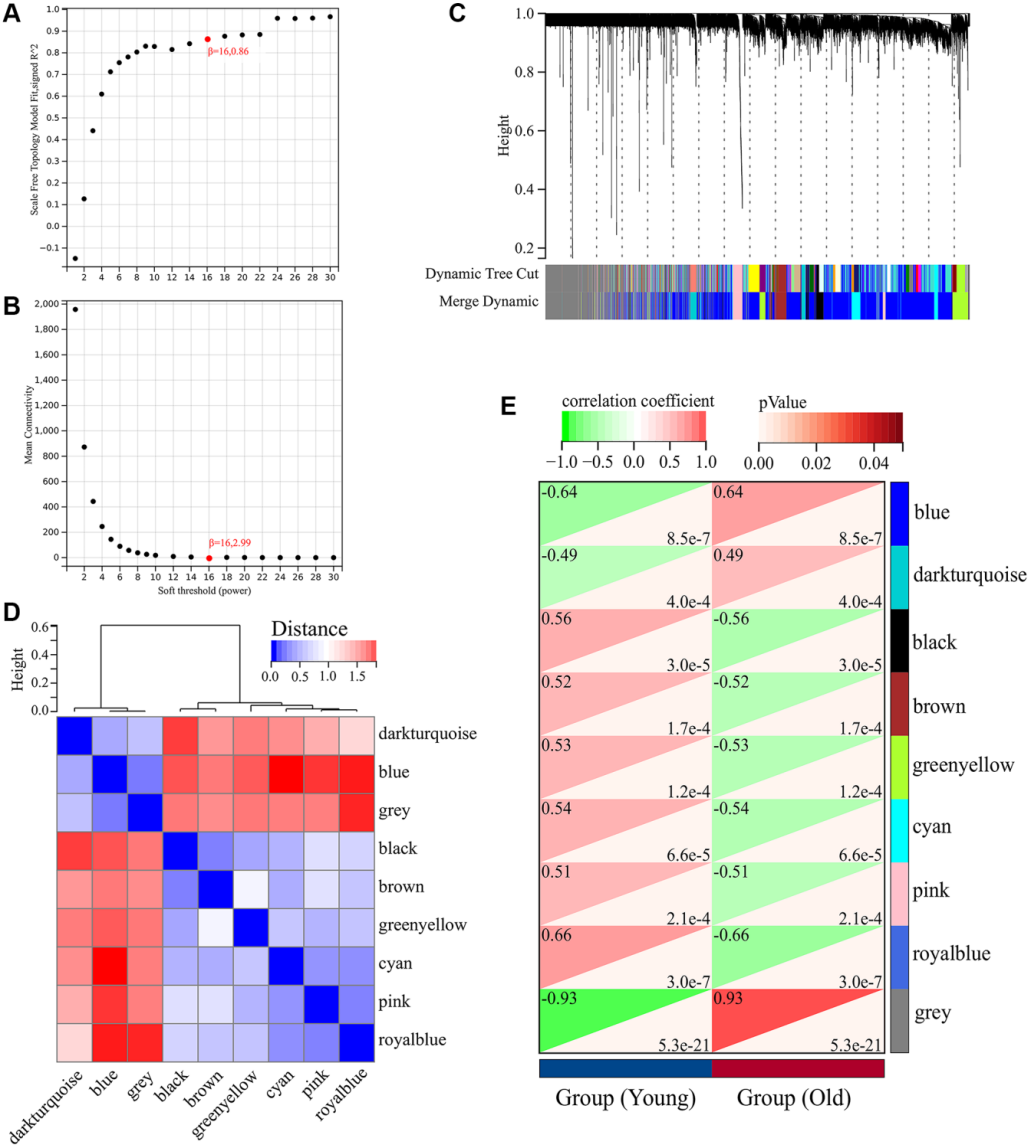
**Weighted gene co-expression network analysis (WGCNA) of DEGs in GSE85358 dataset.** (**A**) Scale independence as a function of soft threshold power. (**B**) Mean connectivity as a function of soft threshold power. (**C**) Cluster dendrogram. Each color represents a specific co-expression module. (**D**) Heat map plot showing the connectivity of module eigengenes (MEs). (**E**) Heat map of the correlation between MEs and clinical traits (Skin aging).

**Figure 3:**
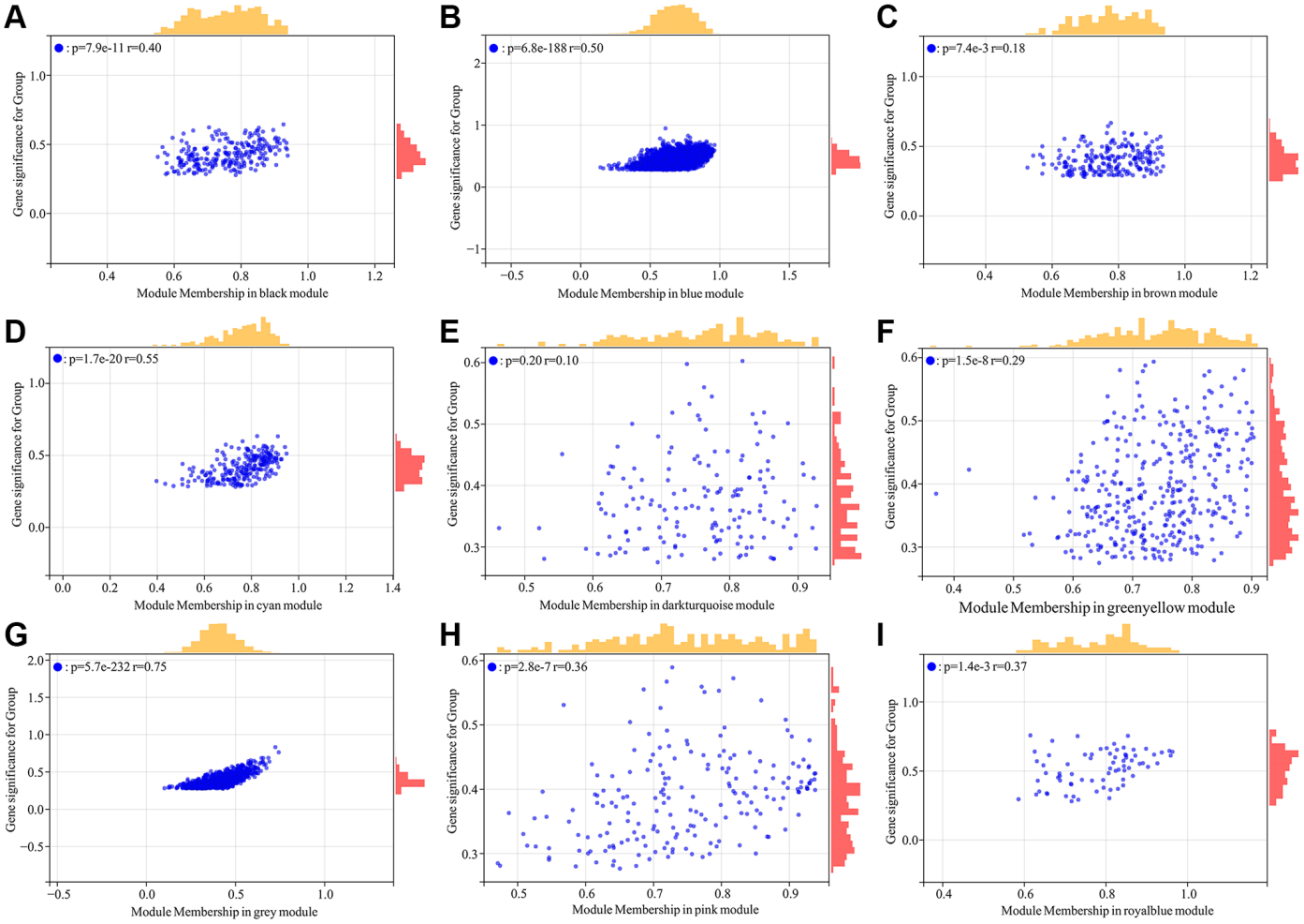
**Identification of hub genes highly associated with skin aging from co-expressed gene modules.** (**A**–**I**) Scatter plot analysis of the module of black, blue, brown, cyan, darkturquoise, greenyellow, grey, pink and royalblue, respectively.

A total of 5726 DEGs were screened from skin tissues of old and young groups in GSE85358, including 3169 upregulated and 2457 down-regulated in the old group (Figure 1A). The expression heat map of DEGs was shown in Figure 1B. The expression matrix of DEGs was used for WGCNA analysis. The WGCNA results were shown in Figures 2 and 3. The optimal soft threshold β was set to 16 (Figure 2A, 2B). Based on the DynamicTreeCut algorithm, with a minimum module size of 30, deep split of 3, and a maximum module distance of 0.25, 9 gene modules were generated, including black, blue, brown, cyan, darkturquoise, greenyellow, grey, pink and royalblue (Figure 2C). We analyzed the connectivity of module eigengenes (MEs), and the results indicated that the distance between the modules was greater than 0.25, indicating that the modules and genes in each module were independent (Figure 2D). The correlation coefficients between these modules and the clinical feature (Skin aging) were calculated. The results showed that all modules were significantly associated with skin aging (Figure 2E). Moreover, according to the criteria of |GS| >0.5 and |MM| >0.5 and *p* value < 0.05, a total of 1162 hub genes highly associated with skin aging were identified from 9 significant modules (Figure 3A–3I).

**Figure 4:**
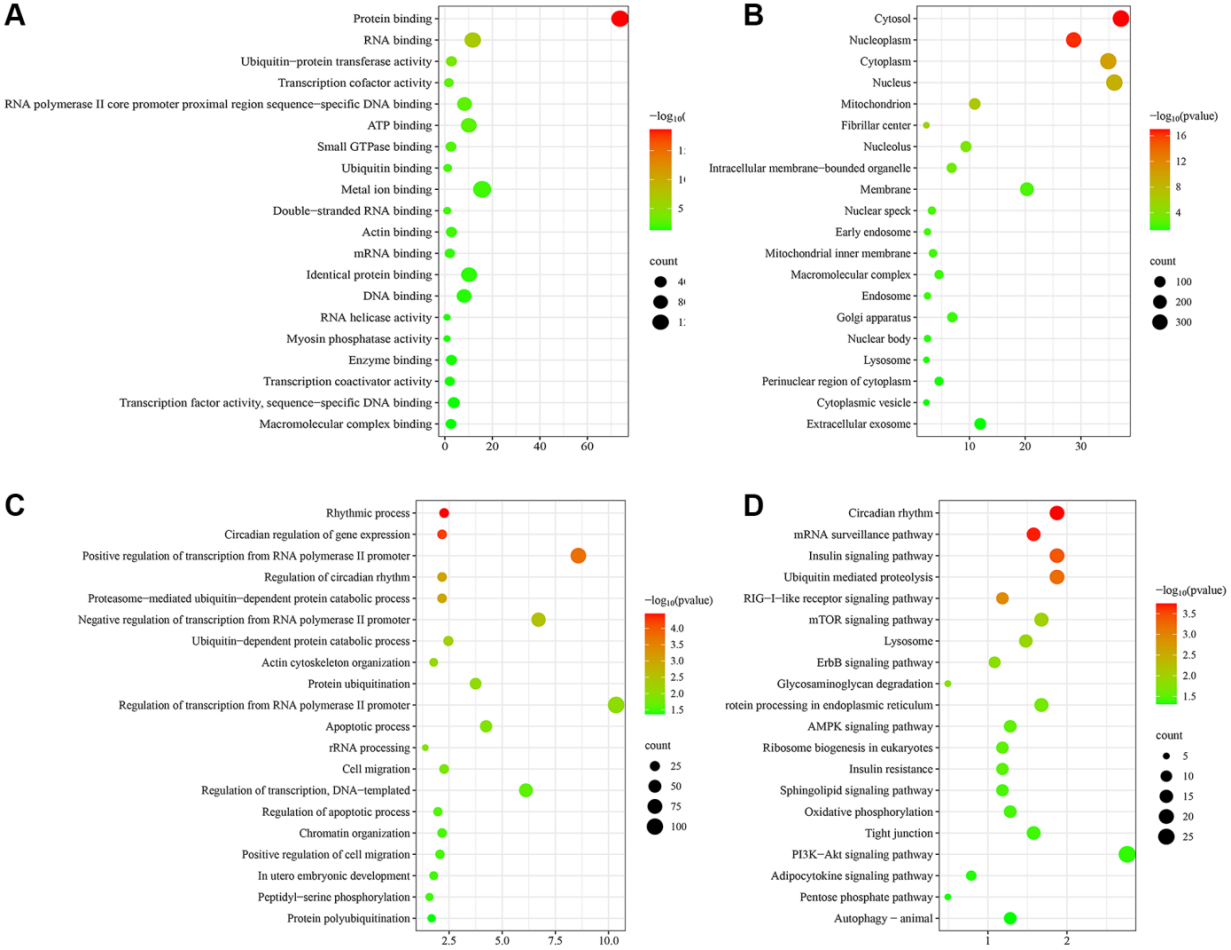
**Functional enrichment analysis of hub genes.** (**A**) Molecular function (MF) analysis. (**B**) Cellular component (CC) analysis. (**C**) Biological processes (BP) analysis. (**D**) KEGG pathway enrichment analysis.

To initially explore the potential molecular mechanisms of skin aging, GO and KEGG functional enrichment analysis of hub genes was performed. The top 20 most significantly enriched terms were displayed in Figure 4. GO analysis suggested that these hub genes exerted multiple functions such as protein binding, RNA binding, ubiquitin-protein transferase activity, transcription cofactor activity, RNA polymerase II core promoter proximal region sequence-specific DNA binding (Figure 4A). The hub genes were mainly enriched in many CCs such as cytosol, nucleoplasm, cytoplasm, nucleus, mitochondrion (Figure 4B). And they were involved in several BPs such as rhythmic process, circadian regulation of gene expression, positive regulation of transcription from RNA polymerase II promoter, regulation of circadian rhythm, proteasome-mediated ubiquitin-dependent protein catabolic process (Figure 4C). KEGG enrichment analysis findings revealed that these hub genes were primarily enriched by signaling pathways including circadian rhythm, mRNA surveillance pathway, insulin signaling pathway, ubiquitin mediated proteolysis, RIG-I-like receptor signaling pathway (Figure 4D). Notably, hub genes were significantly enriched to biological functions and signaling pathways associated with circadian rhythms, suggesting that circadian rhythms may play an important role in the skin aging process.

### Screening of skin aging-related CRGs

**Figure 5:**
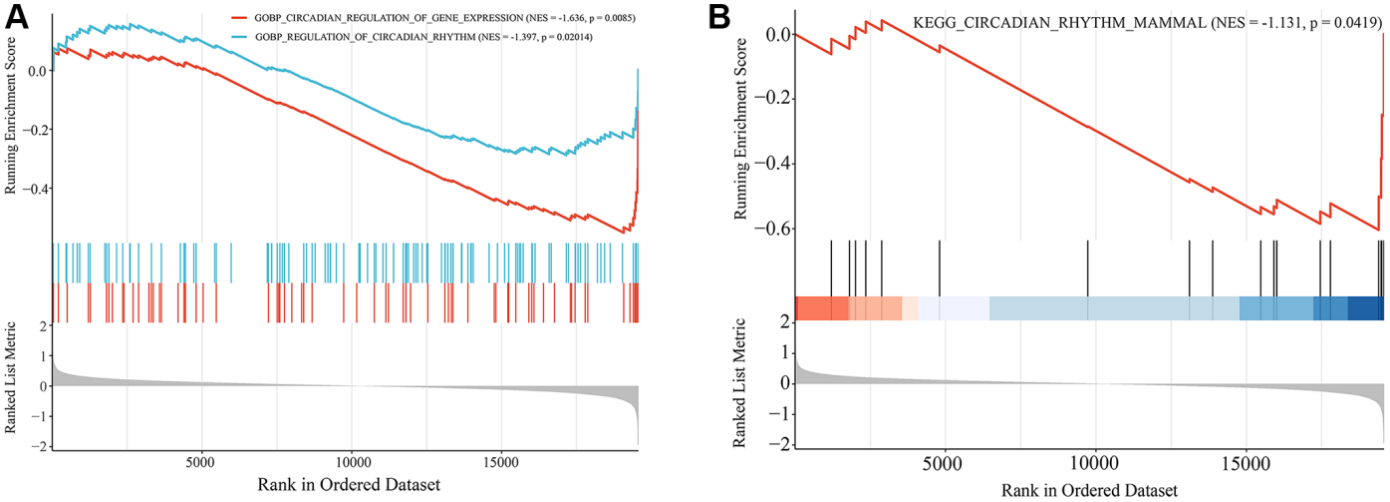
**Gene set enrichment analysis (GSEA) of GSE85358 dataset.** (**A**) Biological processes associated with circadian rhythms. (**B**) KEGG signaling pathways associated with circadian rhythms.

**Figure 6:**
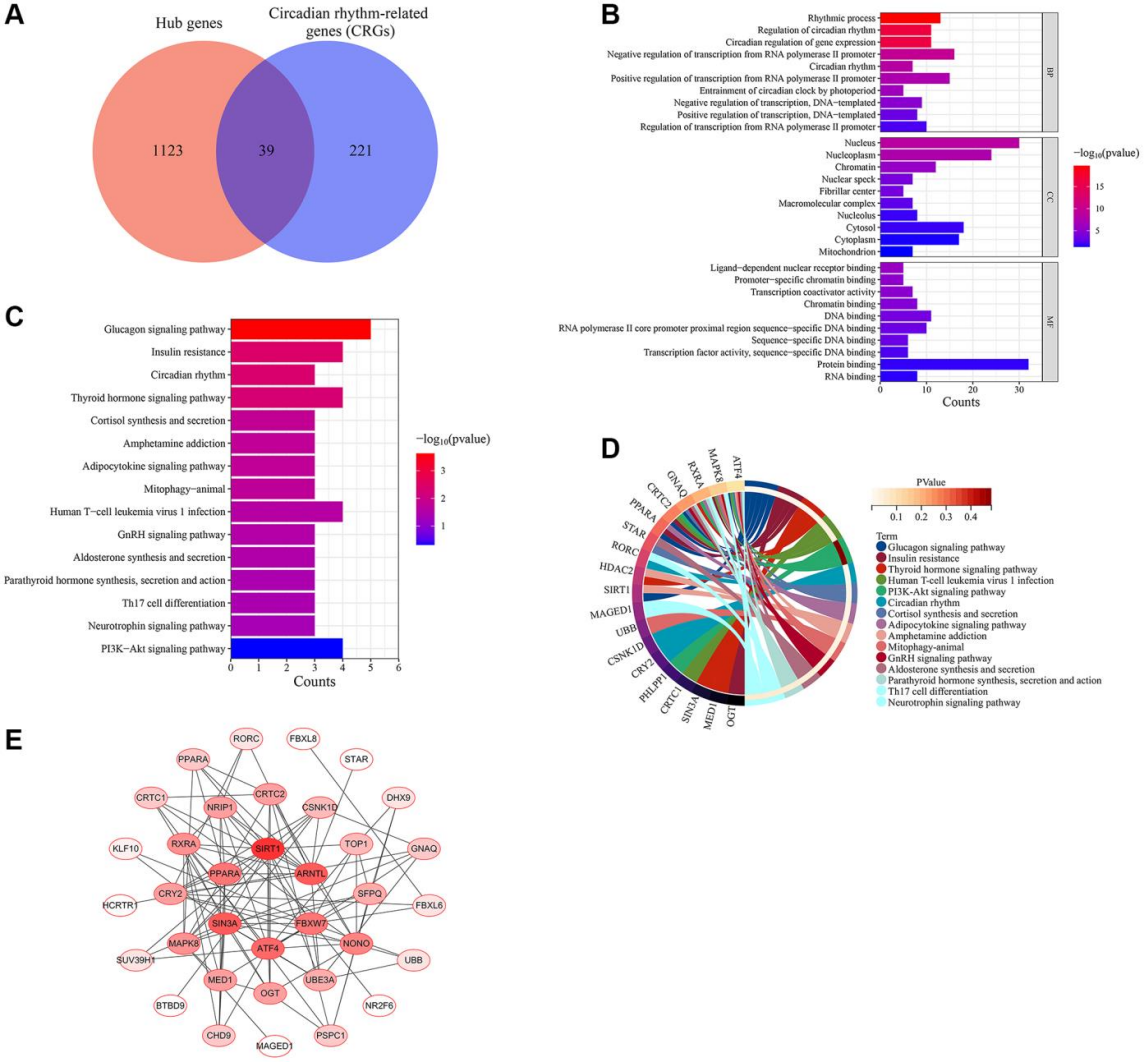
**Screening of skin aging-related CRGs.** (**A**) Skin aging-related CRGs identification. (**B**) GO annotation of skin aging-related CRGs. (**C**) KEGG pathway enrichment analysis of skin aging-related CRGs. (**D**) The interaction of KEGG signaling pathways and their associated CRGs. (**E**) The PPI network of skin aging-related CRGs.

GSEA analysis was used to assess changes in the expression of biological processes and KEGG signaling pathways associated with circadian rhythms in aged skin tissues. The results showed that, compared with the young group of skin tissue, the biological processes and signaling pathways associated with circadian rhythms were significantly downregulated in the old group of skin tissue, according to the GSE85358 dataset (Figure 5A, 5B). This further suggested a strong correlation between circadian rhythms and skin aging. Therefore, we cross-linked the hub gene with CRGs to yield 39 common genes, which are considered skin aging-related CRGs (Figure 6A, Supplementary Table 2). These CRGs mainly participated in various MFs, such as ligand-dependent nuclear receptor binding, promoter-specific chromatin binding, transcription coactivator activity. They were enriched in many CCs including nucleus, nucleoplasm, chromatin, etc. And they were involved in various BPs, such as rhythmic process, regulation of circadian rhythm, circadian regulation of gene expression and circadian rhythm (Figure 6B). The KEGG functional enrichment analysis of skin aging-related CRGs showed that they mainly participated in signaling pathways such as glucagon signaling pathway, insulin resistance, circadian rhythm, thyroid hormone signaling pathway, adipocytokine signaling pathway and PI3K−Akt signaling pathway (Figure 6C, 6D). In addition, the PPI network of skin aging-related CRGs was constructed. As shown in Figure 6E, these genes had a good interaction with each other, and SIRT1, ARNTL, FBXW7, ATF4, SIN3A, and PPARA were the top six genes with the highest degree in the PPI network.

### Screening for key CRGs by machine learning

**Figure 7:**
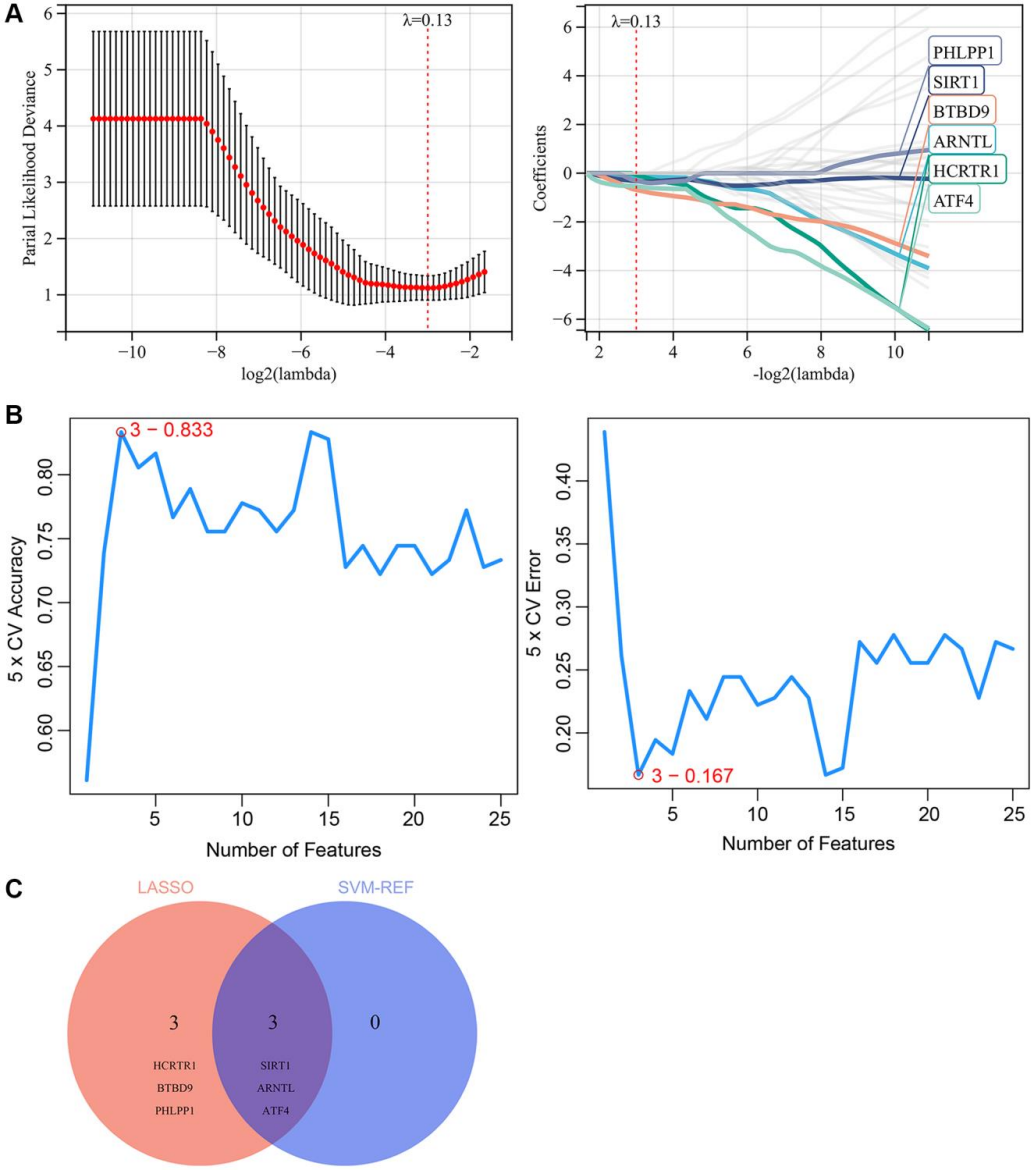
**Screening for key CRGs by machine learning.** (**A**) LASSO regression model screened the potential key CRGs. (**B**) SVM-RFE algorithm screened the potential key CRGs. (**C**) The Venn diagram showed the overlap of key CRGs between the above two machine learning algorithms.

Two machine learning algorithms were used in our study to further screen the key genes from 39 skin aging-related CRGs, which may become potential biomarkers or therapeutic targets for skin aging. Firstly, a LASSO regression model was designed based on samples from aged and young skin tissues. When λ= 0.13, the LASSO regression model was able to accurately evaluate both old and young skin tissues. Therefore, LASSO analysis yielded 6 candidate genes (Figure 7A). Moreover, SVM-REF analysis was performed and identified 3 candidate genes (Figure 7B). Finally, the results from the two algorithms were combined, yielding 3 key CRGs that are associated with skin aging, including SIRT1, ARNTL, ATF4 (Figure 7C).

### Immune infiltration analysis

**Figure 8:**
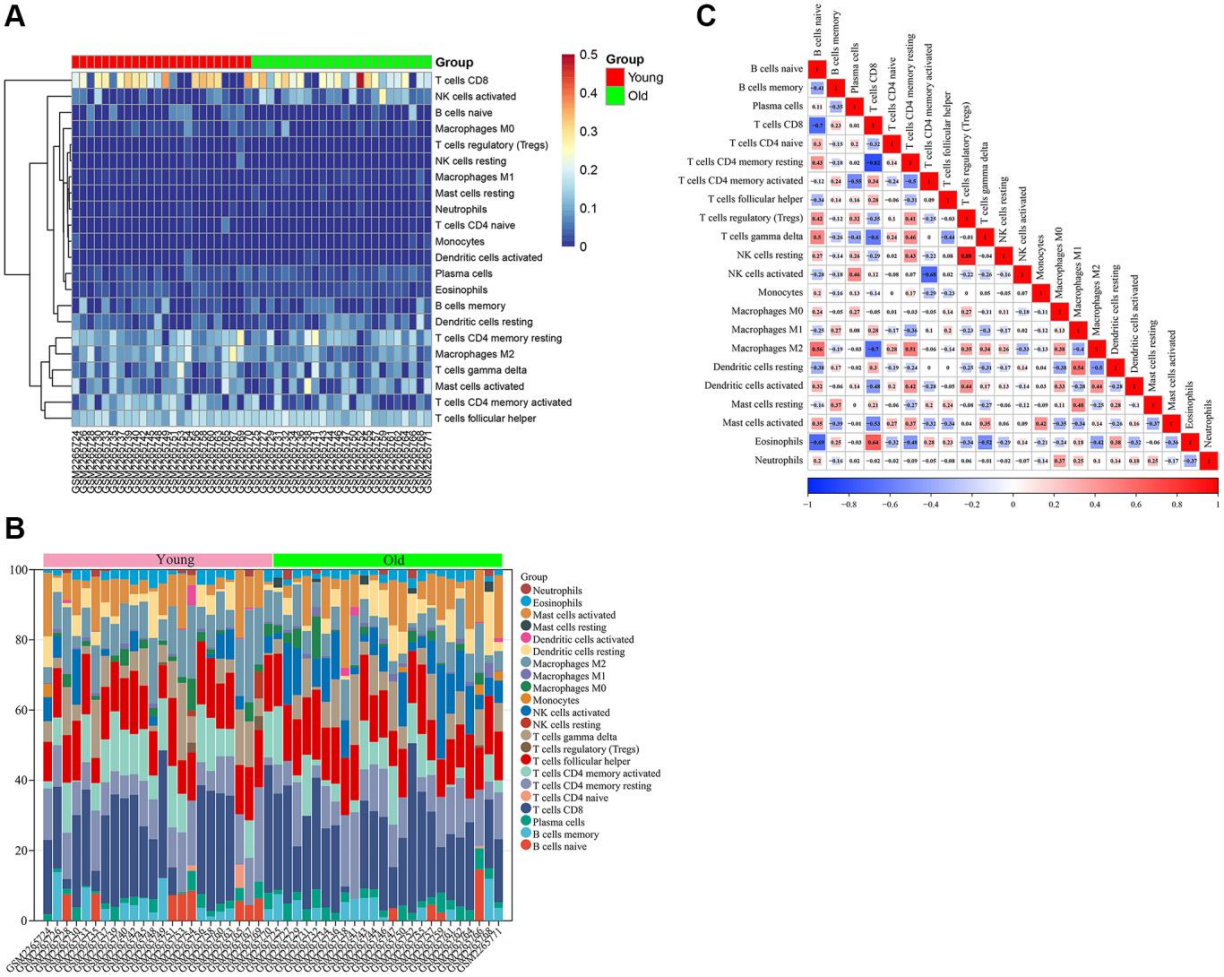
**Immune infiltration analysis of GSE85358 dataset.** (**A**) Heat map of immune cell infiltration in skin tissue of old and young groups. (**B**) Bar graph of immune cell infiltration in skin tissue of old and young groups. (**C**) Correlation between 22 characteristic immune cells in skin samples.

Firstly, we analyzed the infiltration of 22 immune cell types in skin tissues of the young and old groups. The bar graph and heat map showed the type and number of immune cell infiltrates in each sample (Figure 8A, 8B). Subsequently, we assessed the correlation between these immune cell populations (Figure 8C). A strong positive correlation between T cells regulatory (Tregs) and NK cells resting (r = 0.89) and a strong negative correlation between T cells CD8 and T cells CD4 memory resting (r = −0.82) were found.

**Figure 9:**
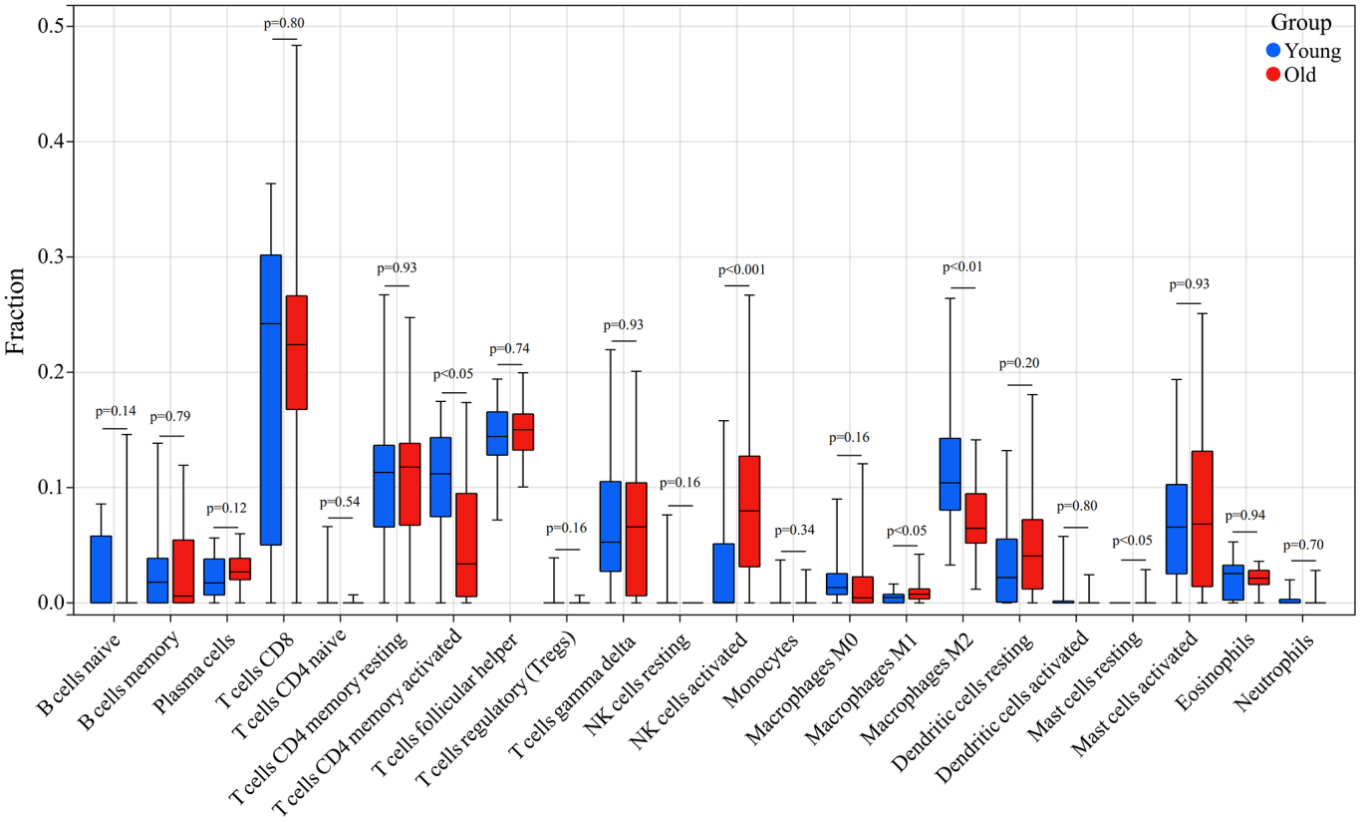
**Comparison of the infiltration of 22 immune cell types in the skin tissue of the old and young groups.** Red indicated the old group and blue indicated the young group; *p* < 0.05 was considered a significant difference.

In addition, we compared the infiltration levels of 22 types of immune cells in skin tissues between the young and old groups. Immune cells such as NK cells activated, Macrophages M1 and Mast cells resting had a significantly higher expression in skin tissue in the old group, whereas T cells CD4 memory activated and Macrophages M2 showed a significantly lower expression in skin tissue in the old group (Figure 9).

**Figure 10:**
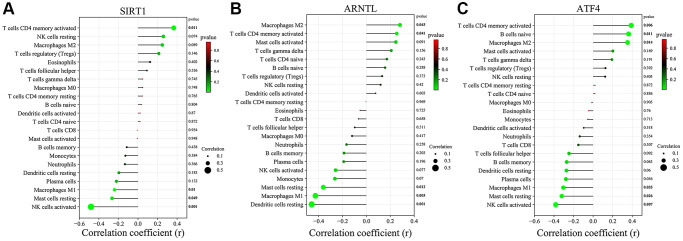
**Correlation of key CRGs in skin tissue with immune cell infiltration.** (**A**) SIRT1. (**B**) ARNTL. (**C**) ATF4.

Furthermore, we further investigated the correlation of 3 key CRGs with 22 immune cell infiltration (Figure 10A–10C). SIRT1 expression showed a positive link to the infiltration status of T cells CD4 memory activated and negatively correlated with the infiltration status of Macrophages M1, Mast cells resting and NK cells activated. ARNTL expression showed a positive link to the infiltration status of Macrophages M2 and T cells CD4 memory activated and negatively correlated with Mast cells resting, Macrophages M1 and Dendritic cells resting. Moreover, ATF4 expression was positively linked to the infiltration status of T cells CD4 memory activated, B cells naive and Macrophages M2 and negative correlation with the infiltration status of Macrophages M1, Mast cells resting and NK cells activated.

### Expression of key CRGs

**Figure 11:**
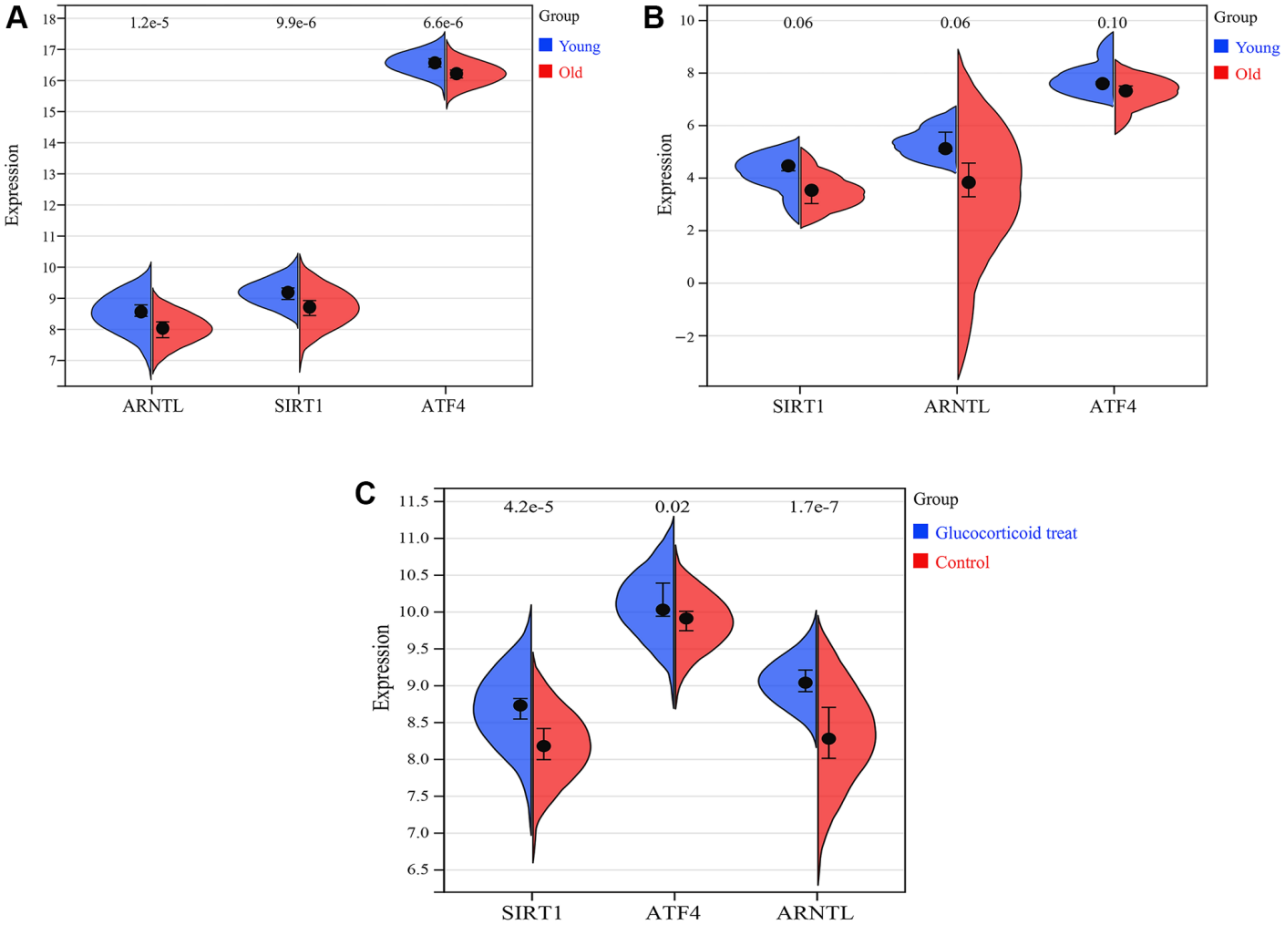
**Expression of SIRT1, ARNTL and ATF4.** (**A**) Expression of SIRT1, ARNTL and ATF4 in training set GSE85358. (**B**) Expression of SIRT1, ARNTL and ATF4 in validation set GSE39170. (**C**) Expression of SIRT1, ARNTL and ATF4 in validation set GSE120783.

We further investigated the expression profiles of SIRT1, ARNTL and ATF4 in skin aging. The results showed that in the training set GSE85358, the expression of SIRT1, ARNTL and ATF4 mRNAs were significantly downregulated in the skin tissue of the old group compared to the young group (Figure 11A). Similarly, in the validation set GSE39170, the expression of SIRT1, ARNTL and ATF4 mRNAs were down-regulated in the skin tissues of the old group compared to the young group. Glucocorticoids are the most commonly used drugs for anti-skin aging in clinical practice (Figure 11B). In the validation set GSE120783, glucocorticoid treatment was used to mimic anti-skin aging. In GSE120783, the expression of SIRT1, ARNTL and ATF4 mRNA was significantly up-regulated in the glucocorticoid treated group compared to the control group (Figure 11C).

### Receiver operating characteristic (ROC) analysis

**Figure 12:**
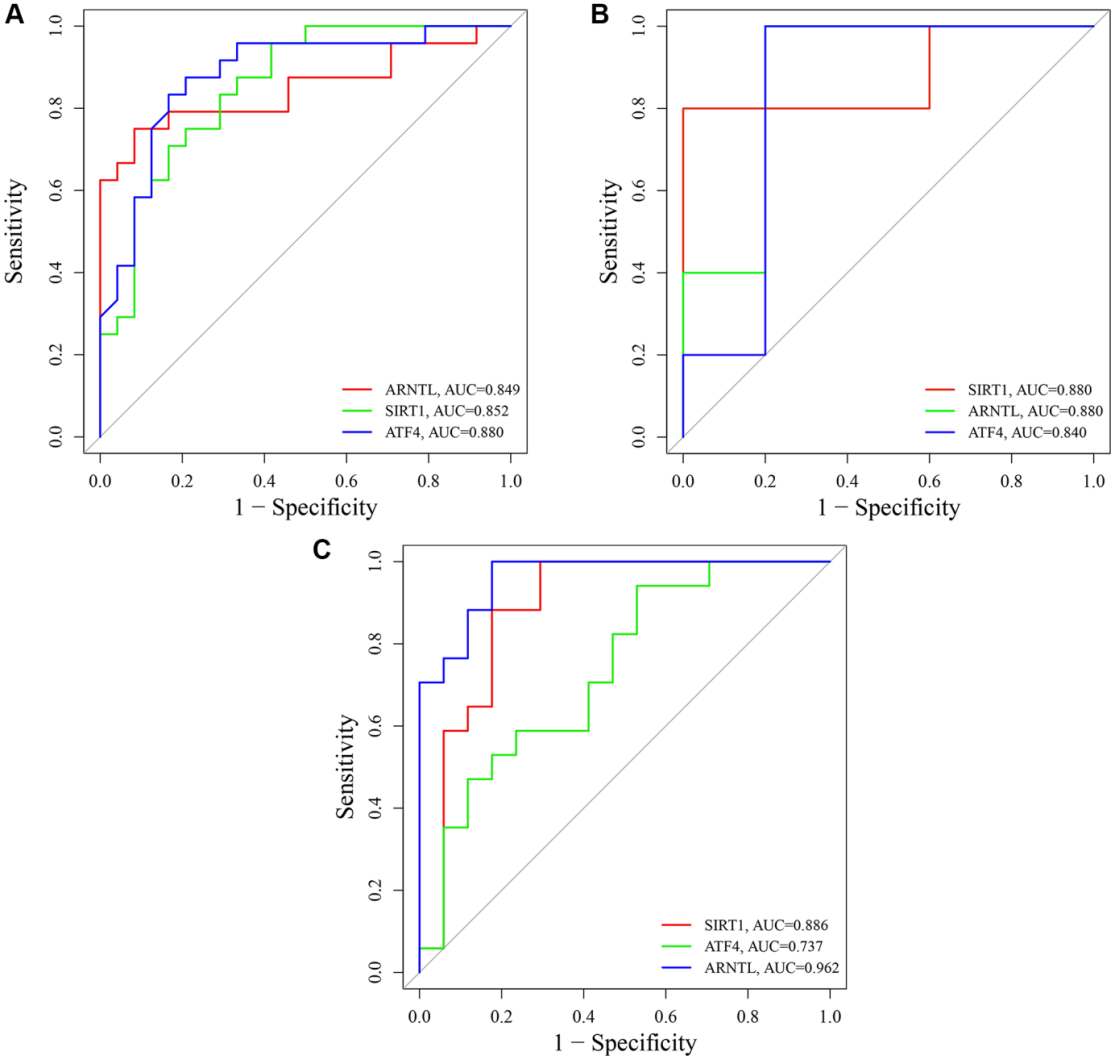
**Receiver operating characteristic (ROC) analysis of SIRT1, ARNTL and ATF4.** (**A**) ROC curves of SIRT1, ARNTL and ATF4 in training set GSE85358. (**B**) ROC curves of SIRT1, ARNTL and ATF4 in validation set GSE39170. (**C**) ROC curves of SIRT1, ARNTL and ATF4 in validation set GSE120783.

To evaluate the diagnostic specificity and sensitivity of key CRGs, we constructed ROC curves and calculated the AUC for each item. In the training set GSE85358, the AUC values of SIRT1, ARNTL, and ATF4 were 0.852, 0.849, and 0.880, respectively (Figure 12A). In the validation set GSE39170, the AUC values of SIRT1, ARNTL, and ATF4 were 0.880, 0.880, and 0.840, respectively (Figure 12B). Moreover, in the validation set GSE120783, the AUC values of SIRT1, ARNTL, and ATF4 were 0.886, 0.962, and 0.737, respectively (Figure 12C). These results indicate that the expression of SIRT1, ARNTL, and ATF4 genes could effectively distinguish aging skin from young skin.

## DISCUSSION

The circadian rhythm is a natural biological clock that regulates various physiological and behavioral activities of the human body, such as sleep cycle, body temperature, hormone levels, and immune responses, through two systems: the central oscillator and the peripheral oscillators [7]. Disruption of the circadian rhythm has been found to be closely associated with various diseases. For example, circadian rhythm disruption can lead to sleep disorders [11]. Circadian rhythm disruption can have adverse effects on appetite, energy metabolism, and weight control, which can lead to the development of obesity and metabolic syndrome [12]. Circadian rhythm disruption can affect the cardiovascular and endocrine systems, increasing the risk of cardiovascular diseases [13, 14]. In addition, circadian rhythm disorders may promote the occurrence and development of tumors, and increase the occurrence of mental and emotional disorders [15, 16]. Maury E et al. found that disruption of circadian rhythms could lead to premature aging and reduced lifespan and fertility in mice [17].

The skin is the largest organ of the body and the outermost protective layer, and it is one of the most direct reflections of the external environment and the internal state of the body. Under the influence of a variety of external and internal factors, the skin will show different degrees of aging. Studies have shown that aging often occurs with disruption of circadian rhythms, and so does skin aging [10]. Normal circadian rhythm contributes to the physiological function and maintenance of the skin. For example, a normal circadian rhythm can restore skin health by releasing cell repair hormones to repair damaged DNA, thereby reducing or even eliminating damage from aging-causing factors such as UV rays [18]. Pathological mechanisms of skin aging, such as oxidative stress, inflammation, and DNA damage, are mostly influenced by circadian rhythm genes [9, 18]. BMAL1 (Basic helix-loop-helix ARNT-like protein 1) (also known as ARNTL) and CLOCK (Circadian locomoter output cycles protein kaput) are two important genes in circadian rhythm regulation, and their proteins bind to form a transcriptional activation complex that initiates transcription of genes in circadian rhythms, which together regulate physiological rhythms in the body. Joo JH et al. showed that BMAL1 expression was significantly downregulated in UV radiation-treated human keratinocytes and that BMAL1 ameliorated UV radiation-induced DNA damage in human keratinocytes [19]. Sun G et al. suggested that upregulation of BMAL1 could ameliorate UV-induced skin aging in mice by suppressing oxidative stress levels [20]. Sun Y et al. revealed that BMAL1 and CLOCK have important functions in regulating apoptosis and DNA damage response in human keratinocytes induced by UVB radiation [21]. The PER (Period) gene is also an important gene in the regulation of circadian rhythms, and Yeom M et al. showed that downregulation of PER gene expression promotes skin aging by upregulating the expression of MMP-1, an enzyme that hydrolyzes collagen [22].

In the present study, we found that biological processes and KEGG signaling pathways related to circadian rhythm were downregulated in aging skin tissue, similar to previous research reported. This further demonstrates the important role of circadian rhythm in skin aging. Based on bioinformatics analysis, we identified 39 skin aging related-CRGs from young and old skin tissues. These CRGs were involved in glucagon signaling pathway, insulin resistance, thyroid hormone signaling pathway, adipocytokine signaling pathway, and PI3K-Akt signaling pathway in addition to circadian signaling pathway. It suggests that the regulation of circadian rhythm by these skin aging related-CRGs may be related to these pathways. Endogenous skin aging not only represents the biological clock changes of the skin tissues themselves, but also reflects the functional decline of internal organs in the body. Declining pituitary, adrenal, and gonadal functions lead to aging-related phenotypes (including skin phenotypes) and behavioral characteristics [23]. Dysregulation of hormones such as glucagon, insulin, and thyroid hormone can affect skin morphology and function, skin permeability, wound healing, sebum lipid production, and skin cell metabolism [23]. Dysregulation of these hormonal pathways is also closely associated with circadian rhythm disturbances [24, 25]. For example, chronic insulin resistance could interfere with the normal regulation of circadian rhythms because insulin regulates glucose absorption and metabolism in the body, which is critical for maintaining the circadian rhythm [24].

Based on machine learning, we identified three key CRGs closely related to skin aging, namely SIRT1, ARNTL and ATF4. Expression of all three key CRGs was negatively correlated with skin aging. ARNTL, also called BMAL1, as discussed above, has been shown to affect skin aging by regulating circadian rhythms. SIRT1 (silent mating type information regulation 2, homolog 1) is an NAD+-dependent deacetylase that is involved in many biological processes. Studies have shown that SIRT1 is involved in regulating cellular oxidative stress, DNA damage and repair, and cell cycle control in skin cells, thus affecting skin aging [26, 27]. SIRT1 is also involved in the regulation of nuclear chromatin structure and can regulate the expression of clock genes BMAL1 and CLOCK, thereby regulating metabolism and behavioral rhythms [28, 29]. Additionally, SIRT1 can also affect circadian rhythm maintenance by regulating sleep cycles and lipid metabolism [15, 30]. ATF4 (Activating Transcription Factor 4) is an important transcription factor that participates in the circadian rhythm regulation. ATF4 binds to the cAMP response element binding protein (CREB) to regulate the expression of clock genes such as ARNTL (BMAL1), CLOCK, and PER2, thus monitoring the biological rhythm process (such as metabolism, sleep, neuronal activity, DNA repair, antioxidant defense, etc.) [31–33]. Lone A N et al. found that ATF4 expression was downregulated and accompanied by oxidative stress, apoptosis, endoplasmic reticulum stress, and decreased cytoplasmic calcium (Ca^2+^) levels in UV-B irradiated human skin fibroblast cells [34]. Although there are no direct reports of SIRT1 and ATF4 regulating skin aging through circadian rhythms, our study provides further evidence for this molecular mechanism.

In addition, immune factors also play an important role in skin aging. Some endogenous, ectopic or denatured molecules produced by damaged or dead cells and organelles can activate autoimmune responses, thus exacerbating the local and systemic aging process. Immune cells could promote the proliferation, differentiation and metabolism of different cell types in the skin by secreting cytokines. The immune microenvironment is altered in aging skin tissue compared to young skin tissue, and the altered immune microenvironment is one of the key causes of skin aging [35]. Circadian rhythms have been found to be important in influencing the immune microenvironment and immune response in the skin. Geyfman M et al. identified several immune function-related genes in a set of genes regulated by circadian rhythms in mouse skin [36]. Prendergast BJ et al. found that removal of the pineal gland in hamsters eliminated circadian rhythmic changes in antigen-presenting dendritic cell transport in the skin and impaired skin antigen-specific delayed hypersensitivity [37]. Qiang L et al. suggested that deletion of the circadian gene SIRT1 inhibited the recruitment of macrophages, neutrophils, and mast cells, thereby inhibiting skin wound healing [38]. Therefore, it is of scientific importance to further investigate the changes in the immune microenvironment during skin aging and the effects of CRGs on the immune microenvironment. In this study, we found that NK cells activated, Macrophages M1, Mast cells resting, T cells CD4 memory activated, and Macrophages M2 may play an important role in skin aging, and the infiltration of these immune cells also correlated to some extent with the expression of circadian rhythm related genes SIRT1, ARNTL and ATF4.

In summary, this study revealed the important role of circadian rhythm in skin aging. Based on bioinformatics and machine learning techniques, we obtained three key CRGs, SIRT1, ARNTL and ATF4, which are potential risk genes for skin aging and are also associated with the alteration of immune microenvironment in skin aging. This study further investigated the molecular mechanisms of skin aging, which will provide a basis and guidance for anti-skin aging research and drug development.

## Supplementary Materials

Supplementary Tables
