# Supplementary material for: Identification of key circadian rhythm genes in skin aging based on bioinformatics and machine learning
Source: Aging (Albany NY). 2023 Oct 30;15(20):11672–89. doi: 10.18632/aging.205155 (PMC10637791; doi:10.18632/aging.205155)
Supplement: Supplementary Tables [file aging-15-205155-s001.pdf]

## SUPPLEMENTARY TABLES

**Supplementary Table 1. 260 circadian rhythm genes (CRGs) collected from the MSigDB database.**

| | | | | | | |
|---|---|---|---|---|---|---|
| ARNTL2 | GFPT1 | SRRD | JUN | ATF5 | PPP1CB | CIPC |
| CRTC2 | METTL3 | HELZ2 | PRF1 | HOMER1 | NLGN1 | MAPK10 |
| SIRT1 | SLC9A3 | TGS1 | BHLHE40 | NOS2 | RORB | PRKDC |
| CLDN4 | HDAC3 | KLF9 | PPP1CC | TYMS | CRTC3 | BHLHE41 |
| NONO | NCOA1 | PTEN | CSNK1E | PER2 | CIART | NR3C1 |
| ARNTL | AGRP | FBXL17 | NMU | CLOCK | HCRTR2 | FASPS4 |
| NR2F6 | MTNR1A | USP2 | CUL1 | PROX1 | PIWIL2 | PER |
| TOP1 | ID2 | NR1D2 | SKP1 | CARM1 | TOP2A | RIGUI |
| ATF4 | ATG7 | HIF1A | JUND | HS3ST2 | NAMPT | TIM |
| MAPK8 | HNF4A | UBA52 | PRKG2 | NTRK1 | ADIPOQ | TIM1 |
| NRIP1 | PPARG | DRD1 | RBM4 | UTS2 | CSF2 | MAGEL2 |
| BTBD9 | USP7 | KMT2A | DRD2 | PER3 | NKX2-1 | PRKCG |
| PHLPP1 | NCOA2 | RELB | NOCT | CREB1 | RORA | AVP |
| RXRA | AHCY | FBXL22 | DBP | MAPK9 | SERPINE1 | NPAS2 |
| CSNK1D | HDAC1 | ADA | SMARCD3 | RAI1 | CHRM1 | HNRNPU |
| RORC | MTTP | GHRH | CRX | CARTPT | IL6 | NMS |
| CRTC1 | TH | NR1H3 | KCND2 | HTR7 | PAX4 | TPH2 |
| SFPQ | MTA1 | SUV39H2 | PRMT5 | NTRK3 | ATOH7 | PER1 |
| CRY2 | BTRC | KLF15 | CRY1 | UTS2R | PASD1 | TBL1XR1 |
| SIN3A | HNRNPD<br>PPARGC1A | DYRK1A | RBM4B | CREM | TIMELESS<br>MEF2D | DDX5 |
| DHX9 | USP9X | LEP | DRD3 | MTNR1B | AANAT | KDM5A |
| FBXW7 | NCOA6 | SETX | SIAH2 | PTGDS | FAS | PROKR2 |
| SUV39H1 | AHR | FBXL3 | ELOVL3 | CPT1A | MC3R | FBXL12 |
| UBB | MYBBP1A<br>TNFRSF11A | ADCY1 | SREBF1 | RPS27A | SLC6A4 | TP53 |
| MED1 | CCAR2 | GHRHR | CST3 | CAVIN3 | GNA11 | FBXW11 |
| FBXL6 | KDM2A | OPN3 | KCNH7 | ID3 | ADRB1 | NR1D1 |
| MAGED1 | PRKAA1 | TARDBP | PROK1 | PML | KLF10 | GM129 |
| FBXL8 | ZFHX3 | UBC | SIK1 | CRH | PSPC1 | FASPS1 |
| GNAQ | NCOR1 | EGR1 | DRD4 | MTOR | CHD9 | KIAA0347 |
| PPARA | ARNT | LGR4 | NPS | ROCK2 | OGT | USP46 |
| UBE3A | OPN5 | SFTPC | EP300 | CREBBP | PPP1CA | EGR3 |
| HCRTR1 | CDK1 | ADORA1 | TBL1X | CDK4 | HNF1B | SIX3 |
| STAR | PRKAA2 | GHRL | DDC | ID4 | NGFR | ADORA2A |
| HDAC2 | ATF2 | OPN4 | KCNMA1 | OPRL1 | TPH1 | GSK3B |
| HEBP1 | NFIL3 | THRAP3 | PROK2 | DDB1 | PDE6B | FASPS3 |
| NAGLU | ARNT2 | MEF2C | FBXL21P | HNRNPD | CHRNB2 | CHRONO |
| SPSB4 | PPARGC1A | EZH2 | SOX14 | KCNA2 | TIMELESS | NPY2R |
| F7 | | | | | | |

**Supplementary Table 2. 39 skin aging-related CRGs.**

| | | | | | |
|---|---|---|---|---|---|
| NR2F6 | NRIP1 | CLDN4 | SIRT1 | NONO | RXRA |
| CRTC2 | TOP1 | BTBD9 | ARNTL | MAPK8 | PHLPP1 |
| ATF4 | DHX9 | OGT | UBB | GNAQ | HCRTR1 |
| SIN3A | MED1 | KLF10 | SUV39H1 | RORC | HDAC2 |
| CRTC1 | SFPQ | STAR | HEBP1 | FBXW7 | FBXL8 |
| FBXL6 | NAGLU | CSNK1D | MAGED1 | PPARA | UBE3A |
| PSPC1 | CHD9 | CRY2 | | | |
